# *De novo* transcriptome assemblies of four accessions of the metal hyperaccumulator plant *Noccaea caerulescens*

**DOI:** 10.1038/sdata.2016.131

**Published:** 2017-01-31

**Authors:** Daniel Blande, Pauliina Halimaa, Arja I Tervahauta, Mark G.M. Aarts, Sirpa O Kärenlampi

**Affiliations:** 1 University of Eastern Finland, Department of Environmental and Biological Sciences, Kuopio 70210, Finland; 2 Wageningen University, Laboratory of Genetics, Wageningen 6708 PB, The Netherlands

**Keywords:** Plant genetics, Sequence annotation, RNA sequencing, Agricultural genetics

## Abstract

*Noccaea caerulescens* of the *Brassicaceae* family has become the key model plant among the metal hyperaccumulator plants. Populations/accessions of *N. caerulescens* from geographic locations with different soil metal concentrations differ in their ability to hyperaccumulate and hypertolerate metals. Comparison of transcriptomes in several accessions provides candidates for detailed exploration of the mechanisms of metal accumulation and tolerance and local adaptation. This can have implications in the development of plants for phytoremediation and improved mineral nutrition. Transcriptomes from root and shoot tissues of four *N. caerulescens* accessions with contrasting Zn, Cd and Ni hyperaccumulation and tolerance traits were sequenced with Illumina Hiseq2000. Transcriptomes were assembled using the Trinity *de novo* assembler and were annotated and the protein sequences predicted. The comparison against the BUSCO plant early release dataset indicated high-quality assemblies. The predicted protein sequences have been clustered into ortholog groups with closely related species. The data serve as important reference sequences in whole transcriptome studies, in analyses of genetic differences between the accessions and other species, and for primer design.

## Background & Summary

*Noccaea caerulescens*, also known as Alpine pennycress, is a metal hyperaccumulating plant of the *Brassicaceae* family, previously classified as *Thlaspi caerulescens*^1^. Hyperaccumulation is a very rare characteristic in plants, with around 500 species identified^2^. Metal hyperaccumulation was first defined in relation to Ni hyperaccumulation^3^. A Ni hyperaccumulator was defined as a plant that could accumulate Ni in shoots at levels *>*1000 μg g^−1^ of dry weight. Hyperaccumulation has been extended to other metals with metal-specific thresholds. For Zn, levels of 3000 μg g^−1^ are used and for Cd 100 μg g^−1^ (ref. 2). Plant hypertolerance refers to plants that are able to grow under high metal concentrations without showing symptoms of toxicity. Metallophytes, plants that occur on metal-enriched soils, can be obligate and require the presence of a particular metal, or facultative, which can grow with or without the metal present. Only a small subset of metallophytes are metal hyperaccumulators. Accessions of *N. caerulescens* are facultative hyperaccumulators of Ni, Zn and Cd, with Zn hyperaccumulation being a species-wide trait, and Ni and Cd hyperaccumulation population-level traits^4^. *N. caerulescens* is used as a model plant species for studies on heavy metal hyperaccumulation due to its small genome size and the high degree of variation in metal hypertolerance and hyperaccumulation profiles between different accessions^2,5,6^.

Metal hyperaccumulating plants are of interest for several reasons. These include biofortification, where attempts are made to increase levels of nutrients in plants, e.g. Fe and Zn in staple crops^7,8^; phytoremediation, where plants can be used to concentrate polluting or contaminating metals, which can then be removed from the environment^9^ and reducing levels of toxic metals in plants, e.g. Cd in rice^10^.

Here we provide transcriptomes of four commonly studied accessions for which detailed Zn, Ni and Cd accumulation and tolerance data are available^6^. Two calamine accessions, La Calamine (LC) and Ganges (GA), are much more tolerant to Zn and Cd than the nonmetallicolous accession Lellingen (LE) and the serpentine accession Monte Prinzera (MP). Furthermore, the GA accession is a Cd hyperaccumulator, whereas MP is sensitive to Cd but hyperaccumulates Ni. The LE accession is least tolerant to Zn, but also has the most efficient Zn translocation capacity among the four accessions. Overall, the accessions show metal-specific root to shoot translocation rates. These mechanisms may be related to gene expression level^11^, but variation in hyperaccumulation or tolerance may also originate from differences in the protein sequences by, e.g., leading to different metal specificity of a metal transporter protein.

Sequence information available for *N. caerulescens* includes 454-sequencing of the transcriptome of the GA accession^12^ yielding 23725 sequences, and an EST library of 4289 sequences from the LC accession^13^. Genome sequencing of the GA accession is underway. SOLiD sequencing of root transcriptomes of GA, LC and MP accessions has been utilised for gene expression analysis^11^ but not for transcriptome assembly and sequence analysis.

The present data consist of assembled transcriptome sequences of the roots and shoots of the *N. caerulescens* accessions GA, LC, LE and MP grown in hydroponics under optimal Zn and Ni exposure. The transcriptomes have been annotated and clustered into ortholog groups with other closely related plant species. The transcriptome data can be used for genome, whole transcriptome and gene level studies, serving as a reference sequence, and also providing a sequence resource for primer design. The ortholog clustering will support comparative gene level studies for linking protein sequence variation to phenotypes. Assembly and release of annotated transcriptomes from Illumina data for the four accessions will serve as a valuable sequence resource for future studies.

## Methods

### Experimental design

Seeds of the *N. caerulescens* accessions GA, LC, MP and LE were germinated in soil, and plants with eight to ten leaves were rinsed and transferred to 10-l containers filled with half-strength Hoagland solution (modified from Schat *et al.*^14^): 3 mM KNO_3_, 2 mM Ca(NO_3_)_2_, 1 mM NH_4_H_2_PO_4_, 0.5 mM MgSO_4_, 1 μM KCl, 25 μM H_3_BO_3_, 2 μM MnSO_4_, 0.1 μM CuSO_4_, 0.1 μM (NH_4_)_6_Mo_7_O_24_, 20 μM Fe(Na)EDTA. For GA and LC, 10 μM ZnSO_4_, and for MP and LE 2 μM ZnSO_4_ was added. In addition, 10 μM NiSO_4_ was added to MP. MES (2 mM) was added and the pH was adjusted to 5.5 with KOH. The plants were grown in three climate chambers: 20/15 °C day/night, 250 μmol/m^-2^/s, 75% RH, light period 14 h per day. Continuously aerated solutions were changed twice a week. After three weeks, twelve plants of uniform appearance (with approx. 14–16 leaves) were pooled from each chamber to obtain three independent biological replicates (roots and shoots separately), frozen in liquid N_2_ and stored at −80 °C.

### Generation of the datasets

RNA was extracted using RNeasy Plant Mini kit (Qiagen). Adequate RNA quality and quantity of RNA samples was ensured by Bioanalyzer (Agilent) analysis. Library preparation and sequencing were performed at the Weill Cornell Medical College Genomics Resources Core Facility (NY, USA). RNA libraries were prepared using Illumina TruSeq RNA-Seq Sample Prep Kit following manufacturer's instructions. Libraries were multiplexed, pooled and sequenced using the Paired End Clustering protocol with 51x2 cycles sequencing on four lanes of Illumina HiSeq2000 (Data Citation 1).

### Processing of the datasets

**Figure 1:**
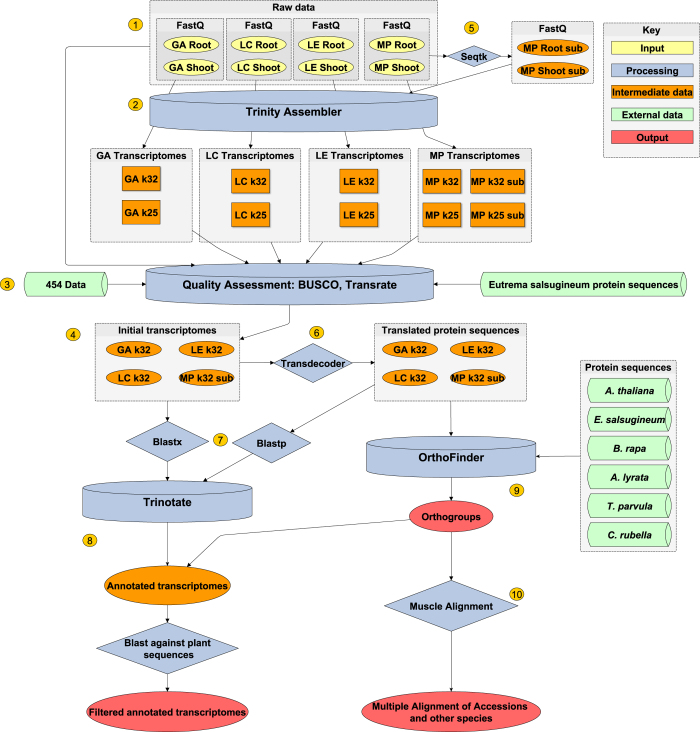
Overview of data processing. Raw reads (1) were assembled using the Trinity Assembler (2) at two kmer values: 25 and 32. Assembly quality was assessed using BUSCO and TransRate (3) utilising external sequence and protein data along with initial raw read sequences. A final assembly was then chosen for each accession (4). For MP accession, reads were also subsampled to the same read depth using seqtk (5) and assembled at both read depths. The predicted protein sequences were obtained using Transdecoder (6). Blast searches were carried out on the protein and transcript sequences against the uniprot and uniref databases (7). These were then combined into an annotation using Trinotate (8). Protein sequences were also clustered into orthogroups using OrthoFinder (9) and protein sequences from other plant species. A multiple alignment was produced from each orthogroup using Muscle (10). Key—Yellow, input data; blue, processing steps; orange, intermediate data/files produced during the process; green, data from public databases; red, final output data.

The overall process for transcriptome assembly, annotation, ortholog clustering and validation is summarised in Fig. 1. After checking the technical quality of the sequencing with FastQC (http://www.bioinformatics.babraham.ac.uk/projects/fastqc/), root and shoot samples for each accession were combined and assembled using the Trinity^15^ *de novo* assembly program at kmer values of 25 and 32. Quality of the assemblies was assessed using BUSCO (ref. 16) (Benchmarking Universal Single-Copy Orthologs) and TransRate^17^. For MP accession with a higher number of reads, subsampling was performed to 105 Million reads using seqtk (https://github.com/lh3/seqtk.git). This step was performed as it has previously been reported that there is an optimum coverage for *de novo* transcriptome assembly^18^. Assembly for MP accession was conducted on both subsampled and complete sets of reads.

Quality of the assemblies was assessed using TransRate and BUSCO. The Kmer 32 assemblies and the MP subsampled kmer 32 assembly were chosen for annotation and ortholog identification. These assemblies are available in the NCBI Transcriptome Shotgun Assembly Sequence Database (Data Citations 2–5). Annotation for each assembly was conducted using the Trinotate program. Orthologs were identified using OrthoFinder. As a final step in the pipeline, each assembly was filtered to remove sequences that did not have a top blast hit to *viridiplantae* (green plant) sequences. After filtering, the BUSCO assessment was performed on the filtered datasets to show whether or not the coverage was reduced.

### *De novo* assembly

Reads for all samples (three biological replicates of both roots and leaves) from each accession were combined, and each accession was assembled separately using the Trinity v2.0.6 *de novo* transcriptome assembler^15^. The total number of reads assembled for each accession is shown in Table 1. The settings that were used for Trinity included quality and adapter trimming using Trimmomatic^19^. No path merging was set so that all sequences with small differences were included in the output. Other settings were kept at default values. Reads were assembled using kmer values of 25 (default) and 32. For the MP accession 219 million reads were sequenced compared to approximately 105 million for the GA, LC and LE accessions. Since it has previously been reported that there is an optimum sequencing depth for transcriptome assembly^18^, we also subsampled 105 million reads from MP using seqtk and assembled these at kmer values of 25 and 32.

### Assessment of assembly quality

The quality of each assembly was checked using TransRate to generate metrics for comparison. The reads generated during the assembly following trimming were provided and used by TransRate to calculate mapping statistics. For the MP subsampled assembly, the complete read files (before subsampling) were used for the mapping. The protein set from *Eutrema salsugineum*^20^ was downloaded from Phytozome 10.2 (ref. 21) and used for TransRate comparative metrics. Assemblies were compared against the BUSCO (ref. 16) plant early release dataset to calculate the extent of coverage (Table 2).

Existing sequences for GA from a 454-sequencing experiment were obtained from the Transcriptome shotgun assembly database GASZ01000000 (ref. 12). These sequences were used for validation and to compare coverage of the assemblies. TransRate and BUSCO quality assessments were performed on this dataset. The highest TransRate scores were obtained for the kmer 32 assemblies and in the case of MP the kmer 32 assembly from sub sampled reads.

### Annotation

The transcripts for each accession for the kmer 32 assemblies were annotated using the Trinotate^15,22–30^ annotation pipeline following the method outlined at (http://trinotate.github.io/). Initially, the transcripts were searched against the custom UniProt and UniRef90 databases using blastx allowing one hit and with output in tabular format. No e-value cut-off was set. The expected protein translations were obtained using TransDecoder and then searched against UniProt and UniRef90 using blastp. The same blast parameters were used as for the blastx searches. The blast searches were loaded into the Trinotate.sqlite database that was obtained from the Trinity ftp site and an annotation report generated. An e-value of 1e-5 was used as the threshold for the blast results during the report generation.

### OrthoFinder

Protein sequences from six other plant species were obtained to identify ortholog groups. *Arabidopsis thaliana* (ATH)^31^, *Arabidopsis lyrata* (ALY)^32^, *Thellungiella parvula* (TPA)^33^, *Brassica rapa* (BRA)^34^ and *Capsella rubella* (CRU)^37^ protein sequences were downloaded from Plaza v 3.0 (ref. 38). *Eutrema salsugineum* (EUT)^20^ sequences were downloaded from Phytozome 10.2 (ref. 21). OrthoFinder^37^ was used to identify groups of orthologs between the species.

### Filtering by top blast hit

As the annotated transcripts could still include non-plant sequences, all transcripts were also searched against the NCBI non-redundant protein sequences (nr) database using blastx and nucleotide collection (nt) database using blastn, both with an e-value cut-off of 1e-5. The blast output format was set as -outfmt ‘6 qseqid staxids sseqid’ to output the taxonomic information for each hit. A python script available in Data Citation 6 was used to parse the taxonomic group information from the NCBI Taxonomy database. Transcripts with a top blast hit to Viridiplantae (‘green plants’) were retained. The fasta files were filtered using cdbfasta (https://sourceforge.net/projects/cdbfasta/) providing the ID of the transcripts to be retained. The BUSCO scores were calculated for the filtered transcript sets to ensure that the assembly coverage was not reduced by the filtering (Table 3). Filtered transcript sequences have been deposited in the NCBI Transcriptome Shotgun Assembly (TSA) sequence database (Data Citations 2–5).

### Multiple alignment

Ortholog groups that contained one or more *N. caerulescens* sequence after top blast hit filtering were retained. The sequences for each group were collected into a fasta file for each individual cluster. Sequences for each cluster were multiply aligned using muscle3.8.31 (ref. 38). Output was selected in fasta and html format. Fasta files and html alignment files for each cluster are available in Data Citation 6.

### Code availability

The python code used to parse taxonomy information is available in Data Citation 6.

## Data Records

The raw sequence data (Data Citation 1 and Table 4) was deposited in the NCBI Sequence Read Archive. The dataset contains 24 records. For each accession (GA, LC, LE and MP) three replicates were sequenced for root and shoot samples. Each replicate was comprised of 12 plants.

The assemblies for each accession at a kmer size of 32 and with subsampled reads for MP (Data Citations 2–5 and Table 5) were deposited in the NCBI Transcriptome Shotgun Assembly Sequence Database.

Full annotation information for the assemblies contained in Excel files and fasta files of ortholog groups (Data Citation 6) are available on Dryad.

## Technical Validation

### Computational Validation

Comparison against the BUSCO plant early release dataset identified that 90 to 91% of single-copy orthologs in the benchmarking dataset were present and complete in the assemblies before and after filtering Tables 2 and 3. TransRate statistics for both mapping and reference based metrics were also high with over 90% of reads mapping to the assemblies and over 80% classed as good mappings Table 2.

### Manual validation of the assemblies

To manually validate the assembly results, complete protein sequences available in Genbank for the accessions were searched. There were results for GA and LC but no sequences were available for LE or MP. In total 14 sequences for GA corresponding to 9 genes and 10 sequences for LC corresponding to 8 genes were analysed. First, a search using blastp was conducted to obtain the matching sequence from the *de novo* assemblies. The sequences were then grouped, where more than one Genbank sequence matched to the same assembled sequence, and a multiple alignment was performed. The similarity of known sequences to the assembly and the length of the alignment was recorded (Table 6). From these sequences, 14 out of 17 had at least 98.9% identity. Sequences that were difficult to assemble from the transcriptome included genes that are known to have multiple copies, e.g. HMA4 (ref. 39)/IRT1 (ref. 40).

## Additional information

**How to cite this article**: Blande, D. *et al.* *De novo* transcriptome assemblies of four accessions of the metal hyperaccumulator plant *Noccaea caerulescens.* *Sci. Data* 4:160131 doi: 10.1038/sdata.2016.131 (2017).

**Publisher’s note**: Springer Nature remains neutral with regard to jurisdictional claims in published maps and institutional affiliations.

## Supplementary Material



## Figures and Tables

**Table 1:** Raw number of reads for each accession.

**Accession**	**Total number of reads**
Ganges (GA)	104697851
La Calamine (LC)	103109619
Lellingen (LE)	105026919
Monte Prinzera (MP)	219339925

**Table 2:** Assembly quality metrics.

										**Subsampled**	
	**GA25**	**GA32**	**454**	**LC25**	**LC32**	**LE25**	**LE32**	**MP25**	**MP32**	**MP25**	**MP32**
*Contig metrics*											
No of transcripts	73,139	40,440	23,725	65,998	37,718	71,508	41,307	108,623	48,400	74,623	46,505
											
*Read mapping metrics*											
% fragments mapped	92	94	82	92	95	92	95	91	86	90	93
% good mappings	82	84	71	83	84	83	84	79	74	79	80
% bases uncovered	24	0	6	25	0	23	0	15	0	15	0
Comparative metrics											
% contigs with CRBB	48	51	76	51	55	46	50	27	44	39	44
% refs with CRBB	60	58	49	60	59	60	59	60	59	59	59
Reference coverage	60	59	37	60	59	60	60	61	59	60	59
*TransRate score*											
TransRate assembly score	0.2343	0.4564	0.3438	0.2367	0.4666	0.2587	0.4607	0.2746	0.3795	0.2755	0.4183
% good contigs	66	80	81	70	77	71	81	75	76	68	75
*BUSCO score*											
% complete	92	90	62	93	91	93	90	93	91	93	90
% duplicated	47	21	18	45	20	44	21	41	23	39	23
% fragmented	1.5	1.4	16	1.6	2.0	1.4	2.4	1.1	1.7	0.9	2.3
% missing	5.5	7.7	20	4.6	6.7	5.2	7.2	5.7	6.9	5.3	7.1
A subset of the assembly quality metrics calculated by TransRate using the trimmed read sequences and *E. salsugineum* protein set, on assemblies constructed at kmer values of 25 and 32. BUSCO score for estimate of assembly completeness.											

**Table 3:** BUSCO quality metrics after assembly filtering.

	**GA**	**GA filtered**	**LC**	**LC filtered**	**LE**	**LE filtered**	**MP**	**MP filtered**
*Contig metrics*								
No of transcripts	40,440	28,885	37,718	28,655	41,307	28,745	46,505	28,599
								
*BUSCO score*								
% complete	90	90	91	90	90	90	90	90
% duplicated	21	20	20	20	21	20	23	22
% fragmented	1.4	1.4	2	2.3	2.4	2.4	2.3	2.1
% missing	7.7	7.9	6.7	6.9	7.2	7.4	7.1	7.4

**Table 4:** Description of samples that have been submitted to the NCBI Sequence Read Archive.

**Sample No**	**Accession/Tissue**	**SRA**	**BioSample**	**Title**
1	GA Root	SRR3742999	SAMN05335705	GA3KR
2		SRR3743000	SAMN05335706	GA4KR
3		SRR3743011	SAMN05335707	GA6KR
4	GA Shoot	SRR3743016	SAMN05335708	GA3KS
5		SRR3743017	SAMN05335709	GA4KS
6		SRR3743018	SAMN05335710	GA6KS
7	LC Root	SRR3743019	SAMN05335711	LC3KR
8		SRR3743020	SAMN05335712	LC4KR
9		SRR3743021	SAMN05335713	LC6KR
10	LC Shoot	SRR3743022	SAMN05335714	LC3KS
11		SRR3743001	SAMN05335715	LC4KS
12		SRR3743002	SAMN05335716	LC6KS
13	LE Root	SRR3743003	SAMN05335717	LE3KR
14		SRR3743004	SAMN05335718	LE4KR
15		SRR3743005	SAMN05335719	LE6KR
16	LE Shoot	SRR3743006	SAMN05335720	LE3KS
17		SRR3743007	SAMN05335721	LE4KS
18		SRR3743008	SAMN05335722	LE6KS
19	MP Root	SRR3743009	SAMN05335723	MP3KR
20		SRR3743010	SAMN05335724	MP4KR
21		SRR3743012	SAMN05335725	MP6KR
22	MP Shoot	SRR3743013	SAMN05335726	MP3KS
23		SRR3743014	SAMN05335727	MP4KS
24		SRR3743015	SAMN05335728	MP6KS

**Table 5:** Description of the Accession numbers for the sequences that have been submitted to the NCBI Transcriptome Shotgun Assembly Sequence Database.

**Assembly**	**Samples**	**Read Samples**	**Accession No**
GA assembly	1–6	SRR3742999SRR3743000SRR3743011SRR3743016SRR3743017SRR3743018	GEVI00000000
LC Assembly	7–12	SRR3743019SRR3743020SRR3743021SRR3743022SRR3743001SRR3743002	GEVK00000000
LE Assembly	13–18	SRR3743003SRR3743004SRR3743005SRR3743006SRR3743007SRR3743008	GEVL00000000
MP Assembly	19–24	SRR3743009SRR3743010SRR3743012SRR3743013SRR3743014SRR3743015	GEVM00000000

**Table 6:** Comparison of assembled sequences to sequences available in Genbank.

**Genes**	**# sequences**	**% pairwise identity**	**Max length**	**Min length**
nicotianamine synthaseGA_TR9812_c0_g1_i1_m.31802 gi|27528464|emb|CAC82913.1|	2	99.7	322	321
ZIP-like zinc transporter ZNT1 GA_TR13622|c0_g1_i1|m.43014 gi|1003366144|gb|AMO45683.1|	2	99.3	408	408
YSL transporter 2GA_TR17962_c0_g1_i1_m.57647 gi|82468793|gb|ABB76762.1| gi|86559333|gb|ABD04074.1|	3	99.8699.86	716	716
YSL transporter 3GA_TR18642_c0_g1_i1_m.60069 gi|82468795|gb|ABB76763.1| gi|86559335|gb|ABD04075.1|	3	99.799.85	672	672
YSL transporter 1GA_TR19192_c0_g1_i1_m.61490 gi|82468791|gb|ABB76761.1| gi|86559337|gb|ABD04076.1|	3	100100	693	693
heavy metal ATPase 4GA_TR19259_c0_g1_i1_m.62343 gi|391225627|gb|AFM38012.1| gi|391225629|gb|AFM38013.1| gi|391225631|gb|AFM38014.1|	4	83.9283.8981.81	1194	1090
heavy metal transporterGA_TR20593_c0_g1_i1_m.68485 gi|66394766|gb|AAY46197.1|	2	100	387	387
hypothetical proteinGA_TR21001_c0_g1_i1_m.69807 gi|91680661|emb|CAI77926.2|	2	86.8	352	349
putative Fe(II) transporter—IRT1GA_TR21885_c0_g1_i1_m.72011 gi|16304676|emb|CAC86382.1|	2	90.1	346	312
ZIP-like zinc transporter—ZNT1 (ATZIP4 homolog)LC_TR1212_c10_g1_i1_m.3330gi|14582255|gb|AAK69429.1|AF275751_1 gi|1003366140|gb|AMO45681.1|	3	10099.51	408	408
metal transporter NRAMP3LC_TR1754_c0_g1_i1_m.5997gi|149688670|gb|ABR27746.1|	2	99.2	512	512
heavy metal ATPase 4LC_TR10517_c0_g1_i1_m.37057gi|391225623|gb|AFM38010.1|gi|391225625|gb|AFM38011.1|	3	98.999.7	1187	1186
ZIP-like zinc transporter ZNT2 (ATZIP4 homolog)LC_TR11232_c0_g1_i1_m.39479gi|14582257|gb|AAK69430.1|AF275752_1	2	100	422	422
nicotianamine synthase 4LC_TR12807|c0_g1_i1|m.44700gi|333733184|gb|AEF97346.1|,	2	100	322	322
chloroplast carbonic anhydrase precursorLC_TR15339_c0_g1_i1_m.51902gi|45451864|gb|AAS65454.1|	2	99.1	336	333
metal transporter NRAMP4LC_TR15506_c0_g1_i1_m.53093gi|149688672|gb|ABR27747.1|,	2	99.6	511	497
zinc transporter—ZTP1 (ATMTP1 homolog)LC_TR19215_c0_g1_i1_m.64186gi|14582253|gb|AAK69428.1|AF275750_1	2	99.7	396	396
Pairwise amino acid identity and the length of the longest and shortest sequence are reported.				
